# Concentrations of uremic bacterial metabolites in patients with post-COVID-19 syndrome

**DOI:** 10.3389/fcimb.2025.1582972

**Published:** 2025-05-29

**Authors:** Natascha Brigo, Wolfram Mayr, Maja Taenzer, Judith Löffler-Ragg, Andrea Schroll, Sabine Engl, Burkhard Schütz, Peter Rappl, Till Heine, Günter Weiss, Katharina Kurz

**Affiliations:** ^1^ Department of Internal Medicine II, Medical University of Innsbruck, Innsbruck, Austria; ^2^ Biovis Diagnostik, Limburg, Germany

**Keywords:** post-COVID-19 syndrome, fatigue, urine metabolome, gut dysbiosis, bacterial uremic metabolites, ME/CFS

## Abstract

Post-COVID-19 syndrome (PCS) is characterized by persistent symptoms and reduced mental and physical performance following the acute phase of COVID-19. The underlying mechanisms remain unclear but may involve gut microbiota dysbiosis and immune-related changes in amino acid metabolism. This pilot study aimed to investigate whether specific bacterial uremic metabolites (BUM) are altered in patients with post-infectious syndromes and whether these alterations are associated with PCS symptoms. We examined BUM in 25 PCS patients, 8 Myalgic Encephalomyelitis/Chronic Fatigue Syndrome (ME/CFS) patients, and 8 healthy controls (Ctrls). Concentrations of BUM were determined in second morning urine samples using mass spectrometry (Biovis Diagnostik, Limburg, Germany). Standardized questionnaires assesed physical, cognitive, psychological, and somatic symptoms and mental health status. PCS and ME/CFS patients exhibited significantly higher scores for post-exertional malaise (PEM) and somatic symptom severity compared to healthy controls (p<0.001). Elevated BUM concentrations were found in 64% of PCS patients, compared to 37.5% of both healthy controls and ME/CFS patients. While overall BUM levels did not significantly differ between groups, heatmap clustering revealed distinct metabolic patterns. Elevated tryptamine and 4-hydroxyphenylpropionic acid (HPHPA) and higher hippuric acid and trimethylamine concentrations, were exclusively analysed in patients with post-infectious syndromes. Our pilot study suggests that urine metabolomic analysis may be a useful approach for investigating the role of gut dysbiosis and BUM in patients with PCS.

## 1 Introduction

Following COVID-19 infection, a significant proportion of patients reports persistent symptoms, collectively termed post-COVID-19 syndrome (PCS). Common manifestations include fatigue, respiratory difficulties, chest pain, and cognitive impairments such as memory and concentration deficits, all of which can significantly impact daily activities. These symptoms may arise anew after initial recovery from acute COVID-19 or persist from the initial illness (Soriano et al., 2021; Kedor et al., 2022).

A notable subset of PCS patients meets the diagnostic criteria for Myalgic Encephalomyelitis/Chronic Fatigue Syndrome (ME/CFS) (Kedor et al., 2022). The symptomatology of ME/CFS—characterized by fatigue, dyspnea, chest pain, chronic cough, anosmia, post-exertional malaise, neurocognitive impairments, anxiety/depression, musculoskeletal pain, lymphadenopathy, headaches, postural tachycardia, and autonomic and neuroendocrine dysfunction—often mirrors PCS (Ianiro et al., 2020; Sahanic et al., 2020; Sonnweber et al., 2020; Nalbandian et al., 2021; Sudre et al., 2021). Emerging research implicates multiple mechanisms in PCS, including gut microbiota imbalances and immune-related disruptions in amino acid metabolism (Paez-Franco et al., 2021; Zhang J. et al., 2023; Raj et al., 2024).

### 1.1 Persistent gastrointestinal symptoms in PCS

Gastrointestinal symptoms such as nausea, vomiting, diarrhea, appetite loss, abdominal pain, and altered taste perception experienced during acute COVID-19 frequently persist afterwards (Yusuf et al., 2021; Gutierrez-Castrellon et al., 2022; Zollner et al., 2022). Studies in clinical and animal models indicate that the intestinal microbiome is involved crucially in on-going gastrointestinal symptoms and broader PCS manifestations (Gutierrez-Castrellon et al., 2022; Sencio et al., 2022; Shen Y. et al., 2022).

The gut microbiota, which varies across individuals and populations, strongly affects human health (Brooks et al., 2018). The composition of the microbiome appears to be critical for conditions such as inflammatory bowel disease (IBD), colorectal cancer, type 2 diabetes, and Parkinson’s disease (Wang et al., 2012; Wallen et al., 2022; Zheng et al., 2022; Li and Guo, 2023), however, precise mechanisms remain unclear until now. Respiratory infections, including COVID-19, also influence the intestinal microbiota via the gut–lung axis (Chunxi et al., 2020; de Oliveira et al., 2021). Recent studies support the hypothesis that microbiome alterations are associated with symptoms of acute COVID-19 and PCS (Brown et al., 2022; Gutierrez-Castrellon et al., 2022).

### 1.2 Microbiota and systemic effects: linking gut, brain, and immunity

The gut-brain axis has gained attention for its bidirectional influence on immune function, neuroendocrine regulation, and mental health. Dysbiosis of the gut microbiota has been implicated in neurocognitive impairments, including those observed in ME/CFS (Mayer et al., 2020; König et al., 2022). Imbalances in the gut microbiome are also associated with a wide range of psychiatric and neurological disorders, including depression, anxiety, and autism spectrum disorders (Mayer et al., 2020).

Intestinal dysbiosis, characterized by a loss of beneficial bacteria and an overgrowth of pathogenic strains, has far-reaching implications for systemic health. Dysbiosis compromises the intestinal barrier, allowing lipopolysaccharides (LPS) to enter the bloodstream. However, not only transfer of LPS to the bloodstream might be problematic, but also the production of toxins like trimethylamine-N-oxide, ammonia, indoxyl sulfate and others (Coulbault et al., 2022). Gut dysbiosis can thereby lead to mitochondrial dysfunction, immune activation, and systemic inflammation, which are implicated in conditions such as obesity, liver disease, autoimmune disorders, and metabolic syndrome (Navaneetharaja et al., 2016; Blomberg et al., 2018; Jackson and Theiss, 2020; Yoo et al., 2020; Leblhuber et al., 2021; Xu et al., 2021; Stolfi et al., 2022). Moreover, these mechanisms affect brain health and mood, thus probably contributing to cognitive and emotional symptoms observed in PCS.

### 1.3 Metabolites linking dietary, microbial, and host metabolic processes

Determination of metabolites produced by gut microbiota can therefore be useful to improve our understanding of the intricate relationship between gut microbiota and host metabolism (Torres-Sanchez et al., 2023). This study focuses on several metabolites of interest, including trimethylamine, trimethylamine-N-oxide, tryptamine, indole-3-acetic acid, indoxyl sulfate, hippuric acid, p-cresol sulfate, 3-(3-hydroxyphenyl)-3-hydroxypropionic acid (HPHPA), and phenylacetylglutamine.

#### 1.3.1 Trimethylamine and trimethylamine-N-oxide

Trimethylamine is generated by bacteria such as *Clostridium* sp., *Escherichia coli*, and *Desulfovibrio* sp. through the enzymatic breakdown of dietary choline, carnitine, and betaine, while trimethylamine-N-oxide is formed when trimethylamine is oxidized in the liver by host enzymes like flavin-containing monooxygenase 3 (Romano et al., 2015; Janeiro et al., 2018; Yang et al., 2019). Trimethylamine-N-oxide, an active dietary metabolite, has been linked to various conditions such as atherosclerosis, hypertension, diabetes, and hyperlipidemia (Wang et al., 2011; Koeth et al., 2013; Tang et al., 2015; Kalagi et al., 2022). Elevated plasma trimethylamine-N-oxide levels also contribute to metabolic disorders and endothelial dysfunction (Shanmugham et al., 2023) and have been associated with symptoms of post-traumatic stress dirsorders in acute myocardial infarction patients (Baranyi et al., 2021).

#### 1.3.2 P-cresol sulfate, hippuric acid and HPHPA

Uremic toxins, such as p-cresol sulfate, hippuric acid, and HPHPA are by-products of protein breakdown mediated by gut microbiota. These compounds significantly contribute to oxidative stress, inflammation, and endothelial dysfunction, which are key factors in the development of atherosclerosis (Dou et al., 2004; Hung et al., 2017). P-cresol is a metabolite of tyrosine and phenylalanine, generated by various intestinal anaerobic and facultative bacteria, including *Clostridium*, *Bacteroides*, *Lactobacillus*, *Enterobacter*, and *Bifidobacterium* species (Sivsammye and Sims, 1990). It is recognized as a cardiovascular risk factor in uremic patients due to its role in promoting reactive oxygen species (ROS), inflammation, and endothelial dysfunction, which contribute to atherosclerosis and thrombosis (Meijers et al., 2008; Chang et al., 2014). Derived from p-cresol, p-cresol sulfate is produced in the gut during bacterial metabolism of aromatic amino acids like tyrosine and phenylalanine (Gryp et al., 2017). Elevated p-cresol sulfate levels are observed in individuals with progressive multiple sclerosis and are linked to worse outcomes in in patients with chronic kidney disease (CKD) (Gryp et al., 2017; Shanmugham et al., 2023).

Hippuric acid is derived from benzoic acid, a microbial degradation product of phenolic compounds, which is then conjugated with glycine in the liver. It serves as a biomarker for fruit and vegetable intake. It has demonstrated protective effects against cholesterol-induced cellular dysfunction through its antioxidant properties and ability to mitigate oxidative stress (Edwards et al., 2022).

HPHPA, an abnormal metabolite of phenylalanine, is formed by gut bacterial metabolism, particularly by anaerobic *Clostridia* species (Shaw, 2010; Kesli et al., 2014). In a case study, elevated HPHPA levels were associated with acute psychosis in a patient with schizophrenia, which improved significantly after treatment with oral vancomycin (Shaw, 2010). HPHPA may act as an amino acid analog of tyrosine and phenylalanine, potentially disrupting large neutral amino acid transport across the blood-brain barrier, influencing neurodevelopment and brain enzyme systems. Overgrowth of *Clostridium difficile*, *Clostridium* sp*orogenes*, and *Clostridium botulinum* is linked to elevated HPHPA levels (Shaw, 2010; Kesli et al., 2014).

#### 1.3.3 Tryptophan metabolites: tryptamine and indole-3-acetic acid

Tryptamine and indole-3-acetic acid are metabolites derived from tryptophan, an essential amino acid integral to neurotransmitter synthesis (serotonin, melatonin) and energy production (Lanser et al., 2020). Tryptamine is produced by *Clostridium sporogenes* and other bacteria via tryptophan decarboxylation, while indole-3-acetic acid is synthesized through pathways involving indole pyruvate and indole lactate, with key contributors like *Escherichia coli* and *Lactobacillus* species (Williams et al., 2014; Roager and Licht, 2018; Tang et al., 2023). Elevated urinary tryptamine levels have been observed in chronic schizophrenia patients, correlating with psychiatric symptoms (Sullivan et al., 1980). Reduced serotonin levels alongside elevated tryptamine levels may contribute to psychosis (Herkert and Keup, 1969).

Indole-3-acetic acid, classified as a uremic toxin at high levels in CKD patients (Duranton et al., 2012) has been linked to autism and the gut bacteria *Clostridia* (Finegold et al., 2002). Conversely, treatment with indole-3-acetic acid in a chronic stress mouse model significantly reduced anxiety and depression-like behaviors (Chen et al., 2022). Additionally, indole-3-acetic acid improved intestinal balance, inhibited inflammation, and restored gut microbiota balance in ankylosing spondylitis mice (Shen J. et al., 2022). Thus, the role of indole-3-acetic acid appears to be worth further exploration, it might have both detrimental or beneficial effects- probably dependent on systemic concentrations and also on host factors like kidney function or environmental stressors.

#### 1.3.4 Phenylacetylglutamine and indoxyl sulfate

Phenylacetylglutamine, a by-product of amino acid metabolism, is closely tied to gut microbiota-mediated amino acid pathways. Similarly, indoxyl sulfate, produced via microbial fermentation of tryptophan, is classified as a uremic toxin (Lauriola et al., 2023).

Indoxyl sulfate is a metabolite of the amino acid tryptophan, produced through dietary intake, digestion, and microbial metabolism in the gut (Huc et al., 2018). It is a by-product of indole metabolism generated by bacteria that produce tryptophanase (Kuo et al., 2021). Protein-derived L-tryptophan is converted into indole by tryptophanase-expressing bacteria in the large intestine. In the liver, the enzyme CYP2E1 metabolizes indole into indoxyl, which is subsequently sulfated by SULT1A1 to form indoxyl sulfate (Lee and Lee, 2010; Roager and Licht, 2018). Elevated urinary indoxyl sulfate levels were linked to *Lachnospiraceae* and *Ruminococcaceae* from the *Clostridia* class, while reduced urinary indoxyl sulfate levels were associated with the *Bacilli* class (Weber et al., 2015). Elevated levels of indoxyl sulfate are associated with CKD-related fatigue and reduced quality of life (Gregg et al., 2021). Mechanistically, indoxyl sulfate disrupts mitochondrial function in muscle cells by increasing glycolysis and pentose phosphate pathway activity while reducing tricarboxylic acid cycle activity, mediated through the nuclear factor erythroid 2-related factor 2 (Nrf2) antioxidative response (Sato et al., 2016). Indoxyl sulfate levels were proposed as gut-specific biomarker in patients of the intensive care unit (Kuo et al., 2021).

Understanding the interconnected roles of dietary metabolism, gut microbial activity, and host biochemical processes in metabolite formation is critical for unraveling the complex interplay between microbiota and host health. Studying the above-mentioned metabolites might not only be useful to assess physiological and pathological states, but might also be useful to monitor effects of microbiota- or diet-directed therapies and metabolic pathways associated with them.

As BUM are produced mainly by pathogenic bacteria in the gut, we wanted to investigate, whether their determination in urine is useful to screen for gut dysbiosis in patients with post-infectious syndromes. As gut dysbiosis can induce inflammation and neuroinflammation (Kearns, 2024) and several BUM have been shown to impair the function of mitochondria and endothelia (Evenepoel et al., 2009; Popkov et al., 2019), we hypothesize that BUM might play a significant role in the pathophysiology of PCS and ME/CFS. Accumulation of BUM could indeed contribute importantly to the development of symptoms like fatigue, neurocognitive deficits, immune system alterations and symptoms of autonomic dysfunction). Thus, our pilot study aimed to investigate whether BUM are altered in post-infectious syndromes compared to healthy controls. It further seeks to determine whether specific metabolites, such as indoxyl sulfate, p-cresol sulfate, trimethylamine-N-oxide, phenylacetylglutamine, trimethylamine, indole-3-acetic acid, HPHPA and hippuric acid, exhibit distinct patterns in post-infectious syndromes. Additionally, the study examines whether BUM concentrations are associated with symptom severity, particularly post-exertional malaise (PEM) and somatic symptoms.

## 2 Materials and methods

### 2.1 Study population

Urine samples were obtained from 32 individuals with post COVID-19 syndrome (PCS), 12 healthy control subjects (Ctrl), and 12 individuals with ME/CFS. PCS and ME/CFS patients were diagnosed and recruited by physicians of the outpatient department for Infectious diseases (Department of Internal Medicine II, University Hospital Innsbruck, Austria). Patients were classified as having PCS if they had persistent symptoms after acute COVID-19 infection for longer than 12 weeks (WHO definition). PCS patients had to have a Post-COVID Functional Status (PCFS) scale score of 2 or higher, indicating moderate to severe functional limitations. The Canadian Consensus Criteria (CCC) were employed to diagnose ME/CFS (further information about the CCC is given in chapter 2.2.1.).

Second-morning urine samples were collected and stabilized with acid between June and December 2022. All participants were instructed—in accordance with the recommendations of Biovis Diagnostik (Limburg, Germany)—not to eat fish or seafood for 2 days prior to urine collection and to avoid cheese, chocolate, nuts, bananas, vanilla, alcohol, caffeine, or nicotine on the evening before urine collection. The second morning urine was collected, because it is a reasonable approximation for the 24 hours collection urine. It provides representative concentrations of BUM such as indoxyl sulfate and p-cresol sulfate without the bias of nocturnal accumulation. Compared to the first morning urine, it is less concentrated and better reflects the daily average, while being more practical and less error-prone than a complete 24 hour collection. This allows a reliable estimation of microbial-induced metabolite accumulation. Urine samples were aliquoted and stored at -80°C until analysis to ensure metabolite stability. For statistical analyses, only the data of patients whose urine creatinine levels were within the reference range provided by Biovis Diagnostik were used. This selection criterion was applied to maintain the integrity of concentration measurements, preventing potential dilution or excessive concentration from affecting the results. Ultimately, only 41 out of 56 patients (73%) were included in the study, as the creatinine levels of the other participants fell outside the specified reference ranges.

Among the study participants, there were 25 individuals with PCS symptoms (20 females and 5 males). Exclusion criteria for the PCS group were individuals under 18 years, pregnant women, patients with dementia, and those unable to provide valid informed consent. Furthermore, participants were excluded if their observation period since infection was less than 12 weeks or their PCFS score was below 2, reflecting low functional impact. These criteria aimed to focus on patients with significant and prolonged post-COVID-19 sequelae while maintaining ethical and methodological rigor.

Eight patients diagnosed with ME/CFS (2 males and 6 females)—which was not due to prior COVID-19 infection—had symptoms including persistent, unexplained chronic fatigue reducing activity levels for at least six months, post-exertional malaise (PEM) as a cardinal symptom, and additional neurological, autonomic, neuroendocrine, or immune symptoms, such as cognitive dysfunction, orthostatic intolerance, or recurrent flu-like symptoms. ME/CFS diagnosis was based on the Canadian Consensus Criteria to ensure diagnostic consistency (further information is given in chapter 2.2.1. Eligible participants had to be 18 years or older and capable of providing informed consent under ICH-GCP guidelines. Exclusion criteria disqualified individuals under 18, those with medical or psychiatric conditions that could explain the symptoms, pregnant or breastfeeding individuals, and those unable to provide informed consent or taking medications or substances that could confound symptom assessment.

Furthermore, a control group consisting of 8 healthy individuals was included for comparison, comprising 7 females and 1 male. The Ctrls were recruited from the staff of the Medical University of Innsbruck. Recruitment was voluntary, and individuals were selected to ensure they had no history of chronic illness, recent infections, or conditions that could interfere with the study’s objectives. Except for one person, all the healthy Ctrls had experienced an acute COVID-19 infection earlier (without hospitalization), but fully recovered and did not have persisting symptoms. Inclusion criteria required participants to report feeling healthy with no current or chronic symptoms, illnesses, or conditions that could indicate underlying health issues. They had to be 18 years or older and capable of providing informed consent according to ICH-GCP guidelines. Individuals with any acute medical conditions or recent infections or post-infectious malaise, or other unexplained symptoms were excluded. Participants had to provide informed consent that complied with ICH-GCP guidelines. Symptom severity was further assessed using validated symptom questionnaires such as DePaul Symptom-Subscale Questionnaire, Patient Health Questionnaire (PHQ), Somatic Symptom Scale (SSS-8), Somatic Symptom Disorder - B Criteria Scale (SSD-12) and The Primary Care PTSD Screen for DSM-5 (PC-PTSD-5). More information about these questionnaires is given below in chapter 2.2.1- 2.2.6).

The study complied with the ethical principles of the Declaration of Helsinki and was approved by the Ethics Committee of the Medical University of Innsbruck (Ethical vote ID: 2017/1157; 1103/2020). Before participation, each patient and healthy Ctrl was fully informed about the study and gave written informed consent.

### 2.2 Participant screening and symptom assessment

The study cohort was given various established questionnaires to provide a comprehensive framework for assessing key symptoms and comorbidities. The Canadian Consensus Criteria (CCC) and DePaul Symptom-Subscale Questionnaire (DSQ-Subscale) are specifically designed for ME/CFS diagnosis and symptom tracking, making them valuable tools for identifying overlapping features in PCS (Bedree et al., 2019; Kedor et al., 2022; Legler et al., 2023; McGarrigle et al., 2024). The Patient Health Questionnaire (PHQ) (Shevlin et al., 2022; Sanal-Hayes et al., 2024), Somatic Symptom Scale-8 (SSS-8) (Honda et al., 2025), and Somatic Symptom Disorder-12 (SSD-12) (Schneider et al., 2023) measure psychological distress and somatic symptom burden, both of which are prevalent in PCS and ME/CFS. Additionally, the Primary Care PTSD Screen for DSM-5 (PC-PTSD-5) addresses trauma-related symptoms, which are increasingly recognized in these populations (Bonazza et al., 2022; Sanal-Hayes et al., 2025). While these questionnaires were previously used primarily for the analysis of ME/CFS, recent studies have applied them to PCS patients and demonstrated strong reliability in chronic illness populations. Together, these instruments provide a multidimensional approach to understanding the symptomatology of PCS and ME/CFS.

#### 2.2.1 Canadian Consensus Criteria

The Canadian Consensus Criteria (CCC) questionnaire was used to evaluate whether participants exhibited symptoms consistent with ME/CFS. The assessment focused on several key symptom domains, including physical and mental exhaustion, the impact of exertion or stress, sleep disturbances, pain, neurological and cognitive manifestations, digestive and urinary symptoms, autonomic dysfunction, neuroendocrine abnormalities, and immunological alterations.

Participants were assessed for unexplained fatigue and exhaustion that significantly reduced activity levels, exacerbation of symptoms after physical or mental exertion (post-exertional malaise), and delayed recovery lasting over 24 hours. Sleep disturbances, such as difficulty falling or staying asleep, altered day-night rhythm, and unrefreshing sleep, were also evaluated. Pain symptoms, including joint pain, muscle pain, and headaches, were included in the analysis. Neurological and cognitive symptoms were assessed through issues such as impaired concentration, memory problems, difficulty processing information, word-finding challenges, sensory sensitivities (e.g., to light or noise), and motor or coordination difficulties. Autonomic symptoms included dizziness or fainting with position changes, heart palpitations, and issues with the temperature regulation like intolerance to heat or cold and abnormal sweating. Additionally, digestive and urinary symptoms included diffuse pain, burning sensations, and bloating. Neuroendocrine abnormalities were identified by heightened sensitivity to stress, emotional instability, appetite or weight changes, and feelings of feverishness or difficulty adapting to temperature changes. Immunological symptoms included painful lymph nodes, recurring sore throats, flu-like symptoms, increased allergies, and hypersensitivity to medications or chemicals.

To meet the criteria for ME/CFS using the CCC, participants were required to fulfill all criteria for physical and mental exhaustion, including post-exertional symptom exacerbation. Additionally, they needed to meet at least one criterion for sleep disturbances, one for pain, and two or more for neurological and cognitive manifestations. Furthermore, they were required to exhibit symptoms from at least two additional domains, including autonomic, neuroendocrine, or immunological manifestations. This rigorous evaluation ensured a comprehensive assessment of ME/CFS symptoms (Carruthers et al., 2011).

#### 2.2.2 DePaul Symptom-Subscale questionnaire

The DePaul Symptom Subscale questionnaire is a tool used to evaluate and assess the intensity and occurrence of symptoms associated with ME/CFS and to measure exercise intolerance. It consists of several questions that cover different aspects of the condition. Participants report on the frequency and severity of their symptoms experienced in the last six months using a five-point Likert scale: 0 (not at all present/symptom absent), 1 (somewhat often/mild), 2 (about half the time/moderately), 3 (most of the time/severe), and 4 (all the time/very severe). Exertion intolerance was assessed by the length of time it took individuals to recover and feel well again after an activity, ranging from 0 hours to 3 days (Bedree et al., 2019). To indicate the presence of Post-Exertional Malaise (PEM), a frequency of at least 2 and a severity of at least 2 must be reported for each of the five questions of the DSQ PEM subscale. However, meeting a frequency of 2 on one question and a severity of 2 on another question does not meet the criteria for PEM. In addition, a minimum of 14 hours is required for a diagnosis of ME/CFS (Bedree et al., 2019).

#### 2.2.3 Patient Health Questionnaire

The PHQ questionnaire is a well-established screening tool that, when combined with a medical consultation, enables an accurate and efficient diagnosis of mental health disorders. The full version assesses conditions such as somatoform disorders, depressive disorders, anxiety disorders, eating disorders, and alcohol abuse. This pilot study specifically examined the subcategories of depression and anxiety, using three targeted questions from the PHQ-9 and the GAD-7 scales. These questions evaluated whether participants had experienced a panic attack within the last four weeks, the severity of these attacks, and whether they had felt persistent nervousness, anxiety, tension, or excessive worry during the same period. A diagnosis of panic syndrome was assigned if participants answered “yes” to all sub-questions in Question 1 and at least four sub-questions in Question 2. For other anxiety syndromes, participants were identified as affected if they affirmed at least four sub-questions in Question 3, provided these symptoms occurred “on more than half of the days in the last two weeks”.

#### 2.2.4 Somatic Symptom Scale (SSS-8)

The 8-item SSS-8 was developed as a brief, patient-reported tool to measure the burden of somatic symptoms. Using a 5-point Likert scale, respondents rate the level of discomfort they experienced from common somatic symptoms over the past seven days. The total score is calculated by summing the ratings, with possible scores ranging from 0 to 32. These scores are then categorized by severity: 0–3 (none to minimal), 4–7 (low), 8–11 (medium), 12–15 (high), and 16–32 (very high). The questionnaire includes the following symptoms: stomach or bowel problems, back pain, pain in the arms, legs, or joints, headaches, chest pain or shortness of breath, dizziness, fatigue or low energy, and difficulty sleeping (Gierk et al., 2014).

#### 2.2.5 Somatic Symptom Disorder - B Criteria Scale (SSD-12)

The SSD-12 questionnaire is commonly used to assess psychological distress in individuals with somatic symptom disorders. It consists of 12 items that evaluate three psychological subdomains of somatic symptom disorders: cognitive, affective, and behavioral, with each domain measured through four specific items. Respondents rate the frequency of their experiences using a 5-point scale: 0 (never), 1 (rarely), 2 (sometimes), 3 (often), and 4 (very often). The total score is obtained by summing the responses, resulting in a range from 0 to 48. A score exceeding 23 points suggests an increased risk for somatic symptom disorder (SSD) (Toussaint et al., 2020).

#### 2.2.6 The Primary Care PTSD Screen for DSM-5 trauma evaluation

The PC-PTSD-5 is a brief, 5-item screening tool designed to identify primary care patients at risk for probable PTSD. The screening begins by assessing lifetime exposure to traumatic events. Respondents who report no exposure are assigned a score of 0. For those who confirm exposure to trauma, particularly within the past year, the questionnaire continues with five additional “yes/no” items that evaluate the impact of the trauma. The final score ranges from 0 to 5, based on the number of affirmative responses to these questions.

### 2.3 Laboratory measurements

Stabilized second morning urine samples underwent analyses conducted by Biovis Diagnostik MVZ GmbH. Liquid chromatography separation was performed on an Agilent 1290 Infinity II with a Restek ARC-18 separation column (2.7 µm, 100x50 mm) for bacterial uremic metabolites. Liquid chromatography solvents were: A- LC/MS grade water with 0.1% formic acid; B- LC/MS grade methanol with 0.1% formic acid and 0.01% trifluoracetic acid. The gradient at a constant flow of 0.4 ml/min was as follows: 0 min 97% A - 0.6 min 97% A - 1.2 min 70% A - 2.4 min 40% A - 3 min 40% A - 3.1 min 20% A - 5 min 20% A - 5.01 min 97% A - 7.5 min 97% A. Mass spectrometry (MS) analysis was performed on a Sciex 5500+ TripleQuad system.

MS settings in positive electro spray ionization mode (ESI+) for indole-3-acetic acid, tryptamine, hippuric acid, phenylacetylglutamine were set as follows: temperature 550°C, ion spray voltage 4500 V, the nebulizer and heat gas pressure 57 psi, curtain gas pressure 39.0 psi and collision gas 8 psi. MS settings in negative electro spray ionization mode (ESI-) for HPHPA, indoxyl sulfate, p-cresol sulfate were set as follows: temperature 550°C, ion spray voltage -4500 V, the nebulizer and heat gas pressure 57 psi, curtain gas pressure 39.0 psi and collision gas 8 psi.

Analyses were performed in Multiple Reaction Monitoring (MRM) mode with a separate isotopically labelled internal standard per analyte (except HPHPA). Notably, the coefficient of variation (CV) for all parameters were below 15%. The CV of the validation are measurement of more than 5 samples on 5 measurement days, each in 5-fold determination; the CVs mentioned are the highest from this validation. The lower limits of quantification (LLOQs) and the upper limits of quantification (ULOQs) are also shown (**Table 1**).

**Table 1:** Reference to the validation parameters of the analytical method (CV, LLOQ and ULOQ).

Parameter	CV	LLOQ [µmol/l]	ULOQ [µmol/l]
Hippuric acid	4.7	2.84	11300
HPHPA	6.35	0.98	2765
Indoxyl sulfate	3.52	0.72	1419
p-cresol sulfate	3.59	0.44	5240
Phenylacetylglutamine	5.66	0.5	1137
Tryptamine	8.22	0.01	1248
Indole-3-acetic acid	5.92	1	456

The high specificity of MS is based on the precise detection of m/z values of the target molecules and the possibility to investigate structure-related fragmentation products by MS/MS analyses. In our method, we use Multiple Reaction Monitoring (MRM) to monitor specific mass transitions (precursor-product ion pairs), minimizing interference from matrix effects. In addition, we use internal standards (stable isotope labelling) for improved analytical accuracy and specificity (**Table 2**).

**Table 2:** Multiple reaction monitoring (MRM) parameters for analytes and internal standards.

MRM+	Q1 Mass [Da]	Q3 Mass [Da]	DP [volts]	EP [volts]	CE [volts]	Retention time [min]
Tryptamine	161.2	144.1	66	12	24	2.9
Indole-3-acetic acid	176.2	130.1	115	10	62	3.75
Hippuric acid	180.2	77.1	38	12	102	3.13
Phenylacetylglutamine	265.1	130.1	90	12	59	3.16
Tryptamine (internal standard)	165.2	148.1	60	12	23	2.9
Hippuric acid (internal standard)	185.2	110	68	12	23	3.13
Indole-3-acetic acid (internal standard)	175.2	132	110	10	62	3.75
Phenylacetylglutamine (internal standard)	269.1	134.1	90	10	55	3.16
MRM-	Q1 Mass [Da]	Q3 Mass [Da]	DP [volts]	EP [volts]	CE [volts]	Retention time [min]
Indoxyl sulfate	212.1	80	-100	-10	-108	2.95
p-cresol sulfate	187	107	-100	-12	-92	3.35
HPHPA	181.1	59	-75	-12	-38	2.96
Indoxylsulfate (internal standard)	218	80	-90	-10	-45	2.95
p-cresol sulfate (internal standard)	194	114	-100	-10	-32	3.35

MRM, Multiple Reaction Monitoring.

Q1 Mass [Da]: Mass-to-charge ratio of the precursor ion (Daltons).

Q3 Mass [Da]: Mass-to-charge ratio of the product ion (Daltons).

DP [volts]: Declustering Potential.

EP [volts]: Entrance Potential.

CE [volts]: Collision Energy.

Reference ranges of the analyzed metabolites were determined internally based on the percentile distribution of n > 100 participants (healthy according to self-report) and are now continuously checked and optimized using statistical methods from meanwhile >10,000 samples. Sample preparation was: 25 µl urine (resp. urine calibrator, quality control) was mixed with 250 µl solvent A and 20 µl internal standard mix. After vortexing and centrifugation, 6 µl of the supernatant was injected into the LCS/MS-MS system. The method for determination of the above-mentioned metabolites was developed by Dipl. Chem. Till Heine of Biovis Diagnostik.

### 2.4 Statistical analysis

Continuous variables were summarized as medians with interquartile ranges (IGR; 25th – 75th percentile) due to their non-normal distribution, as determined by the Shapiro-Wilk test. Categorical variables were presented as frequencies (n) and percentages.

Comparisons of continuous variables across more than two independent groups were conducted using the Kruskal-Wallis test and pairwise group comparisons were performed using Mann-Whitney U test as a *post hoc* analysis. For two-group comparisons of continuous variables, the Mann-Whitney U test was directly applied. Fisher’s exact test was used to test independence of groups comparing categorical variables with small cell sizes (n < 5). P-values were adjusted for multiple testing using the Benjamini-Hochberg method correcting the false discovery rate (fdr) at 5% per symptom category.

Subgroup analyses were conducted to evaluate differences in metabolites in the presence and absence of symptoms. To visualize relationships between variables, a heatmap with hierarchical clustering was performed on rank-transformed data. The clustering was based on Euclidean distance and used the Ward’s D2 linkage method. Clustered data were presented as percentiles to standardize comparisons.

Statistical analyses and visualizations were conducted using R statistical software (Version 4.2.1, R Core Team 2021) using the packages tidyverse, gtsummary, DescTools, Hmisc and pheatmap, and GraphPad Prism (Version 10.1.2).

## 3 Results

### 3.1 Demographic data and inflammatory markers of the study population

The healthy control (Ctrl) group and post-COVID-19 syndrome (PCS) group consisted primarily of females (87.5% and 80.0%, respectively), while the Myalgic Encephalomyelitis/Chronic Fatigue Syndrome (ME/CFS) group consisted primarily of males (75.0%) (**Table 3**), reflecting a significant difference in sex distribution across the three analyzed groups (p = 0.011).

**Table 3:** Descriptive statistics of the study population.

Characteristic	Ctrl, N = 8^1^	PCS, N = 25^1^	ME/CFS, N = 8^1^	p-value^2^
Sex				0.011
male	1 (12.5%)	5 (20.0%)	6 (75.0%)	
female	7 (87.5%)	20 (80.0%)	2 (25.0%)	
Age, years	42 (30, 46)	39 (32, 52)	39 (37, 45)	>0.9
Body mass index, kg/m^2^	22 (18, 30)	24 (18, 30)	24 (21, 25)	0.8
CRP, mg/dL	0.07 (0.06, 0.12)	0.06 (0.06, 0.15)	0.06 (0.06, 0.06)	0.4

^1^n (%); Median (IQR; 25th -75th percentile) for Age, BMI and CRP.

^2^Fisher’s exact test; Kruskal-Wallis rank sum test.

The age distribution across the groups was comparable, with a median age of 42 years (IQR: 30–46) in the Ctrls, 39 years (IQR: 32–52) in the PCS cohort, and 39 years (IQR: 37–45) in the ME/CFS group, demonstrating no significant differences (n.s.) (**Table 3**).

Similarly, median body mass index (BMI) did not show significant differences (p = 0.8), with values of 22 (IQR: 18–30) for the healthy Ctrls, 24 (IQR: 18–30) for the PCS cohort, and 24 (IQR: 21–25) observed for the ME/CFS group (**Table 3**). Inflammation parameter C-reactive protein (CRP) was considered normal when below 0.5 mg/dl, which was found in nearly all patients (except for 2 PCS patients with 0.51 mg/dl and 1.54 mg/dl). CRP levels did not differ between Ctrls and patients with post-infectious syndromes (**Table 3**).

These findings underscore substantial sex disparities among the cohorts, with age, BMI, and CRP levels remaining constant.

### 3.2 Questionnaires

Results of established questionnaires assessing physical, cognitive, psychological, and somatic symptoms, mental health disorders, and trauma impacts (**Table 4**). The Kruskal-Wallis rank-sum test identified significant differences in the median scores of the questionnaires across the Ctrl, PCS, and ME/CFS groups. This result suggests that at least one group differs from the others. To further analyze these differences, we conducted pairwise group comparisons using a Mann-Whitney U test and corrected for multiple comparison using the Benjamini-Hochberg method.

**Table 4:** Scores of questionnaires.

Characteristic	Ctrl, N = 8^1^	PCS, N = 25^1^	ME/CFS, N = 8^1^	p-value^2^
PEM	0 (0, 0)	27 (22, 33)	31 (20, 36)	**0.002**
SSS-8	4 (3, 6)	15 (9, 18)	16 (9, 18)	**<0.001**
SSD-12	0 (0, 2)	24 (15, 28)	25 (22, 29)	**<0.001**
PTSD-Score	0.00 (0.00, 0.00)	0.00 (0.00, 0.00)	0.00 (0.00, 0.00)	0.5
PHQD	4.0 (1.0, 4.3)	8.0 (2.0, 17.0)	3.0 (2.0, 5.0)	0.2
GAD-7	3.0 (1.0, 4.0)	4.5 (2.0, 8.0)	3.0 (2.0, 5.0)	0.2

^1^Median (IQR; 25th -75th percentile).

^2^Kruskal-Wallis rank sum test.

Significant P-values are bold.

PEM scores were significantly higher in the PCS group (median: 27, IQR: 22–33) and ME/CFS group (median: 31, IQR: 20–36) compared to the Ctrl group (median: 0, IQR: 0–0), with a p-value of 0.002 and p-value of 0.005, respectively. SSS-8 scores were also markedly elevated in the PCS group (median: 15, IQR: 9–18) and ME/CFS group (median: 16, IQR: 9–18) compared to the Ctrl group (median 4, IQR: 3–6), with a p-value of 0.001 and p-value of 0.002, respectively. Similarly, SSD-12 scores were significantly higher in the PCS group (median: 24, IQR: 15–28) the ME/CFS group (median: 25, IQR: 22–29) compared to the Ctrl group (median: 0, IQR: 0–2), with a p-value of 0.003 and 0.003, respectively. PTSD scores were consistently low across all groups and showed no significant differences (p = 0.5). PHQD and GAD-7 scores did not differ among groups.

Our findings highlight significant differences in somatic symptom-related measures, with the PCS and ME/CFS groups exhibiting notably higher scores compared to the control group. This observation indicates a pronounced burden of physical symptoms and their associated severity in these conditions. In contrast, the absence of significant differences in PTSD, depression, and anxiety scores suggests that while psychological symptoms may play a role, they are not distinguishing factors between these groups in this study.

**Table 5** presents the results of Fisher’s exact test, which was used to compare the presence of symptoms based on the CCC across the three groups (Ctrl, PCS, and ME/CFS). The Fisher’s exact test identified significant differences in the proportions of participants responding “Yes” or “No” to the various CCC subgroups, as indicated by a significant p-value. This result suggests that at least one group has a different distribution of responses compared to the others.

**Table 5:** Fisher’s exact test results comparing symptom prevalence across Ctrl, PCS, and ME/CFS groups based on CCC.

Category CCC	Symptom	p-value^1^	p-value adjusted^2^
Sleep	Sleep does not lead to recovery	**0.011**	0.061
Pain	Headaches	**0.001**	**0.012**
Neurological and cognitive symptoms	Concentration impairment	**0.002**	**0.013**
Neurological and cognitive symptoms	Processing difficulties	**<0.001**	**0.004**
Neurological and cognitive symptoms	Word-finding issues	**0.037**	0.2
Neurological and cognitive symptoms	Reading difficulties	**0.015**	0.071
Neurological and cognitive symptoms	Overload symptoms	**0.011**	0.061
Neuroendocrine abnormalities	Stress sensitivity	**<0.001**	**0.007**

^1^Fisher’s exact test.

^2^Benjamini & Hochberg correction for multiple testing.

Significant P-values are bold.

The unadjusted p-values showed several significant differences among the groups, especially regarding neurological and cognitive symptoms. After adjustment for multiple testing, symptoms such as concentration impairment (p = 0.002), processing difficulties (p < 0.001), and overload symptoms (p = 0.002) as well as stress sensitivity (p = 0.007) and headache (p = 0.006) were detected in patients with post-infectious syndromes (i.e. PCS and ME/CFS) more often. Conversely, symptoms such as word-finding issues (p = 0.32) and overall neurological and cognitive symptoms (p = 0.419) lost significance after adjustment.

Cognitive dysfunction, manifesting as processing difficulties and concentration impairment, emerged as a prominent feature in PCS, suggesting neurological involvement that may be linked to neuroinflammation or autonomic dysfunction. Furthermore, headaches and sensory overload symptoms indicate disturbances in pain processing and heightened sensitivity, which may be associated with mitochondrial dysfunction. Sleep disturbances, particularly non-restorative sleep, further support the presence of autonomic and circadian rhythm disruptions. Collectively, these results reinforce the overlap between PCS and ME/CFS symptoms.

### 3.3 Bacterial uremic metabolites

**Figure 1:**
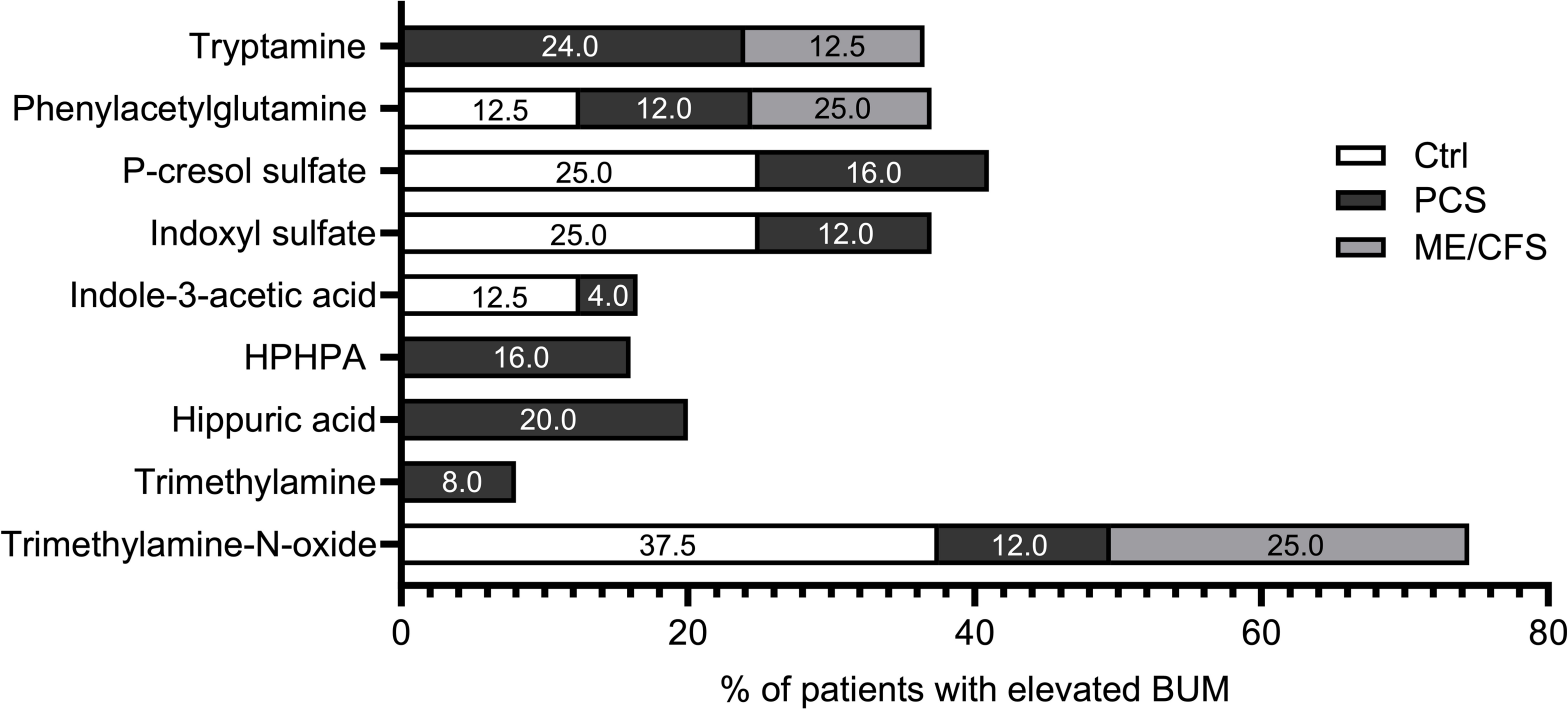
Percentage of elevated bacterial uremic metabolites. Percentage of subjects with elevated bacterial uremic metabolites calculated in each study cohort: Ctrl (white), PCS (black), and ME/CFS (grey). Each bar represents the percentage of participants in each group with elevated concentrations of the specific metabolite.

In patients with PCS, elevated concentrations of at least one bacterial uremic metabolite were detected more often (64%) than in Ctrls (37.5%) and ME/CFS (37.5%); however, Fisher’s Exact test did not provide evidence of significant differences. Of note, elevations of two specific bacterial metabolites were exclusively found in PCS subjects: HPHPA and hippuric acid, while tryptamine was elevated in both PCS and ME/CFS but not in Ctrls. Trimethylamine was only elevated in two participants of the PCS cohort (**Figure 1**).

To visualize the differences in elevated metabolites, we calculated the percentage for each metabolite in each study cohort (8 Ctrls, 25 PCS, and 8 ME/CFS).

Differences in the prevalence of elevated BUM among PCS, ME/CFS, and healthy controls are illustrated in **Figure 1**. PCS patients showed the highest frequency of elevated metabolites, particularly trimethylamine (8%), hippuric acid (20%), tryptamine (24%) and HPHPA (16%), which are linked to altered gut microbial metabolism and systemic inflammation. ME/CFS patients also presented with increased levels of specific metabolites, such as phenylacetylglutamine (25%) and trimethylamine-N-oxide (25%), further supporting metabolic disturbances in these conditions. The presence of elevated trimethylamine-N-oxide (37.5%) in healthy controls suggests individual variability in gut microbiota composition but at a lower prevalence of other BUMs compared to PCS and ME/CFS.

**Figure 2:**
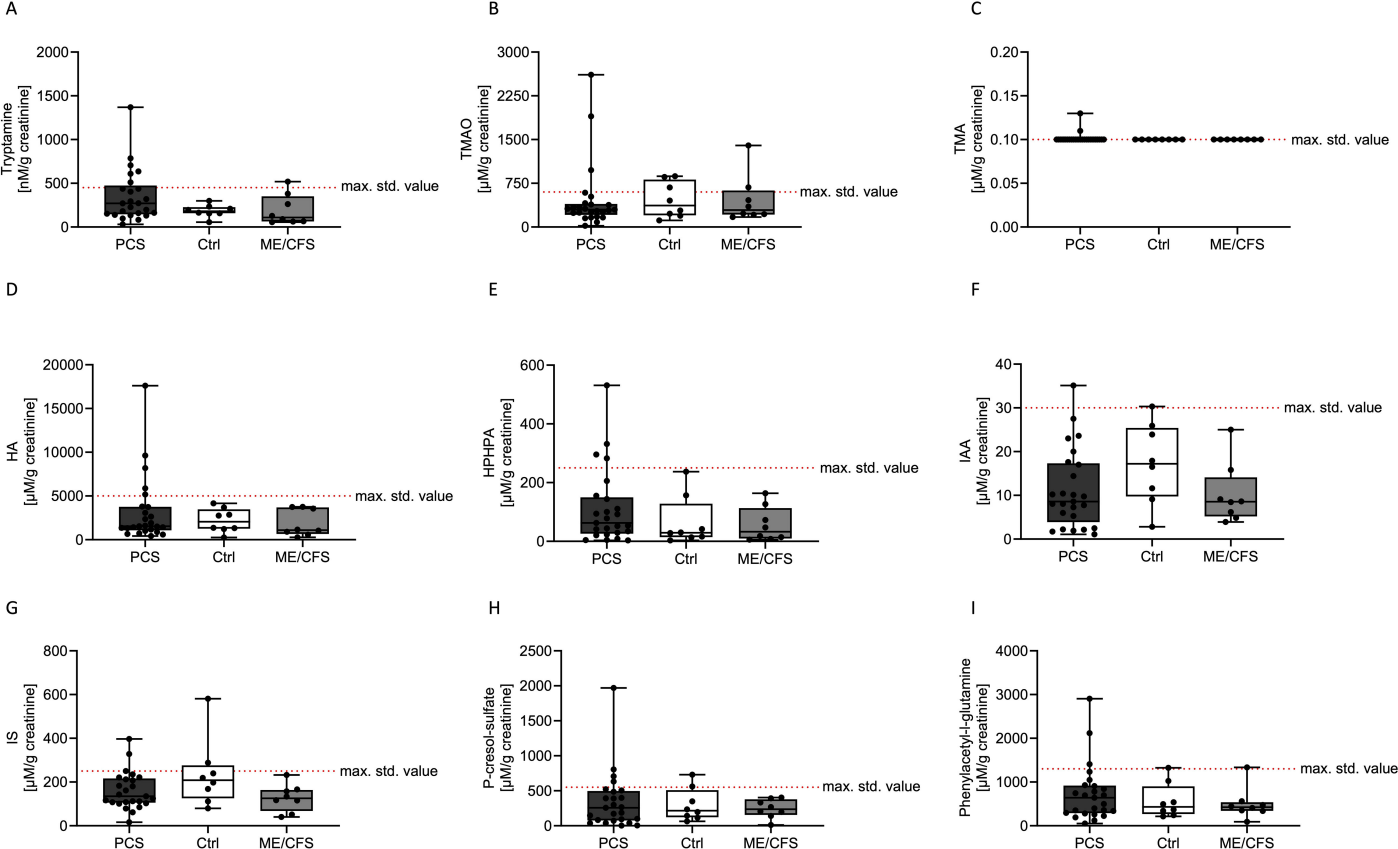
Concentrations of various bacterial uremic metabolites (tryptamine; **A**), trimethylamine-N-oxide (TMAO; **B**), trimethylamine (TMA; **C**), hippuric acid (HA; **D**), HPHPA **(E)**, indole-3-acetic acid (IAA; **F**), indoxyl sulfate (IS; **G**) p-cresol sulfate **(H)** and phenylacetylglutamine **(I)**) in urine samples of patients with post COVID-19 syndrome (PCS, black), healthy controls (Ctrl, white) and myalgic encephalitis/chronic fatigue syndrome (ME/CFS, grey). The maximum standard value of n > 100 participants (healthy according to self-report) based on the percentile distribution are depicted as a dashed red line (max. std. value). Data are presented as box and whisker blots, where each symbol represented a measurement from an individual in the study population.

The concentrations of all the investigated single BUMs did not differ between the Ctrl, PCS, and ME/CFS groups for any of the measured metabolites (**Figure 2**).

Tryptamine levels were found to be lowest in the Ctrl group, moderately elevated in ME/CFS, and highest in the PCS cohort (**Figure 2A**). Trimethylamine-N-oxide levels exhibited a similar trend, increasing from Ctrl to PCS and reaching a peak in ME/CFS (**Figure 2B**). Trimethylamine concentrations appeared to be relatively similar, with two elevated levels in the PCS cohort (**Figure 2C**). Hippuric acid levels were higher in the Ctrl cohort compared to PCS and ME/CFS, but 5 participants in the PCS cohort showed elevated levels (**Figure 2D**). HPHPA levels were higher than the reference range only in the PCS group, while ME/CFS exhibited slightly lower levels than PCS (**Figure 2E**). Indole-3-acetic acid levels were highest in the Ctrl group, however, the levels of the PCS cohort were spread (**Figure 2F**). The concentrations of indoxyl sulfate were highest in the Ctrl group and moderately elevated in the ME/CFS group, with the lowest levels observed in the PCS group (**Figure 2G**). P-cresol sulfate and phenylacetylglutamine concentrations were elevated in the PCS group more often compared to the other groups, with intermediate levels observed in the ME/CFS group and the lowest levels in the Ctrl group (**Figures 2H, I**).

**Figure 3:**
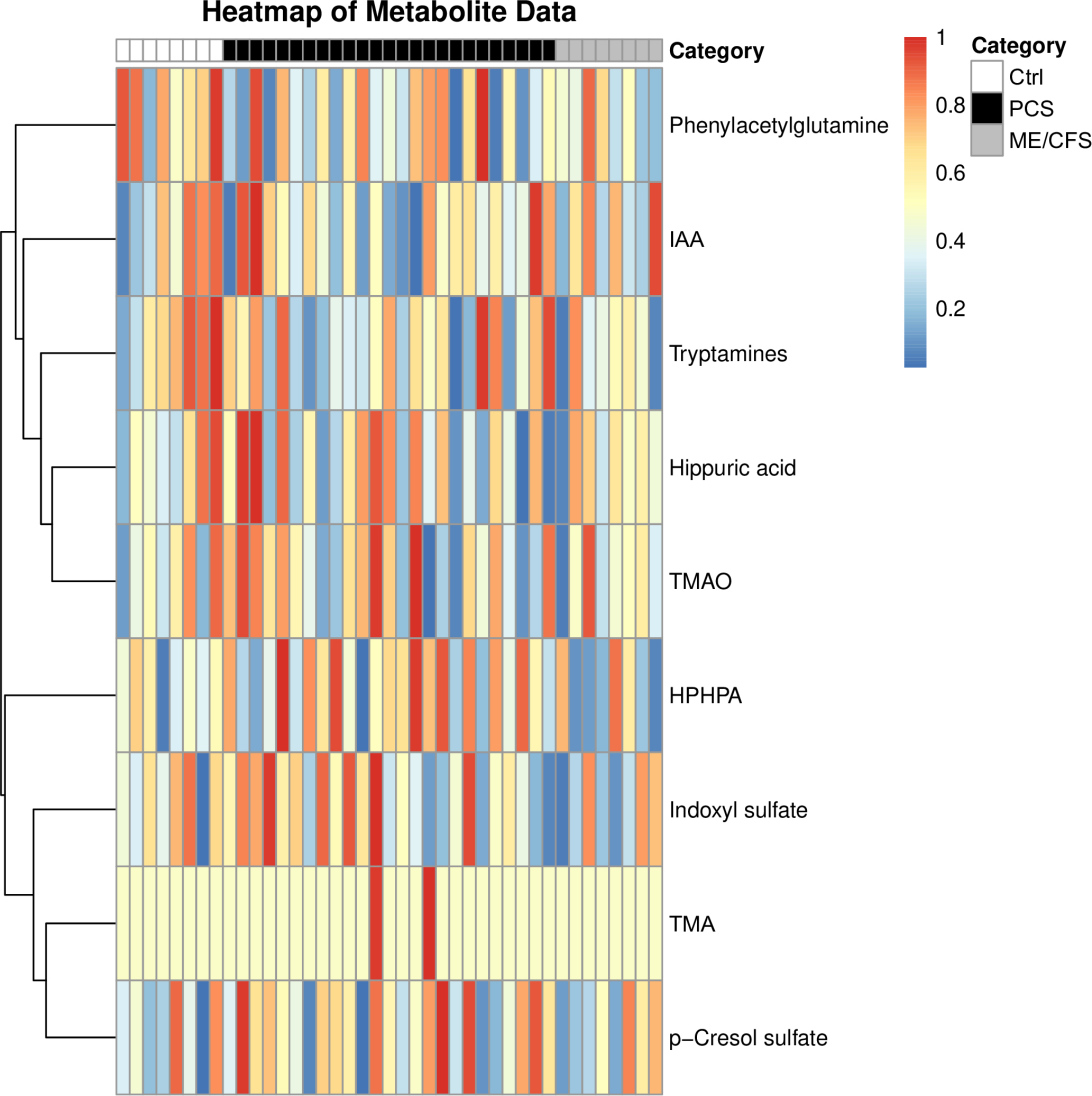
Heatmap analysis of bacterial uremic metabolites within the study population. Each row represents one individual metabolite, while columns represent study participant. Metabolites in the percentile above 50% are depicted in red, and metabolites in the percentile below the 50% in dark blue. The intensity of each color reflects the degree of difference compared to the median value of each metabolite. This allows for intuitive visualization of metabolic variation across study groups, highlighting potential metabolic imbalances associated with disease states. The analyzed bacterial uremic metabolites were: phenylacetylglutamine, indole-3-acetic acid (IAA), tryptamines, hippuric acid, trimethylamine-N-oxide (TMAO), HPHPA, indoxyl sulfate, trimethylamine (TMA) and p-cresol sulfate.

Variations of urinary concentrations of BUMs across PCS, ME/CFS, and healthy controls appeared to reflect key metabolic differences, but also suggested individual differences in microbiome composition and metabolite clearance. A heatmap was created following the comparative analysis of the various metabolites to visually represent and further explore the metabolic differences across the Ctrl, PCS, and ME/CFS groups, providing an overview of clustering patterns and emphasizing group-specific variations in metabolite profiles (**Figure 3**).

The heatmap provides a visual representation of the relative abundance of key metabolites across the three groups: Ctrl, PCS, and ME/CFS. It is accompanied by dendrograms, which cluster the metabolites on similarities in their metabolic profiles. The clustering patterns illustrate potential shared or distinct metabolic pathways in the study population, revealing key metabolic signatures that may differentiate disease states. Given that many of these metabolites are influenced by gut microbial activity, the heatmap also indirectly reflects alterations in gut microbiota composition and function. As microbial metabolites are also metabolized by the liver, kidney, and leaky gut is triggering systemic inflammation, determination of these BUMs can also provide insights into e.g. the function of detoxifying organs and thus also help to understand the pathophysiology of PCS and ME/CFS.

The Ctrl group is representative of baseline metabolic profiles, thus serving as a reference for the purpose of comparison with the PCS and ME/CFS groups. Metabolites such as indole-3-acetic acid, trimethylamine-N-oxide, and indoxyl sulfate were observed at stable levels, reflecting balanced microbial metabolism, liver function, and renal clearance. The stability of these metabolites suggests proper microbial homeostasis and efficient metabolic processing, which may be disrupted in disease states. Any deviations in these metabolite levels within the PCS and ME/CFS groups may signal underlying metabolic dysfunction. The PCS group exhibited pronounced alterations in metabolite levels, reflecting significant disruptions in metabolic pathways. Notably, metabolites such as p-cresol sulfate, tryptamines, and trimethylamine-N-oxide showed significant elevations, pointing to dysregulation of the gut-liver axis, increased microbial fermentation activity, and oxidative stress. These disruptions indicate a potential shift towards a pro-inflammatory and toxic metabolic state, which may contribute to disease progression. Other metabolites, such as indole-3-acetic acid and indoxyl sulfate, exhibited slightly reduced levels with some study cohort participants having significantly increased levels of these two metabolites. This heterogeneity suggests that post-infectious fatigue syndromes may present with diverse metabolic phenotypes, potentially influenced by differences in gut microbiome composition, immune activation, and liver detoxification capacity.

The ME/CFS group displayed metabolic disturbances similar to PCS, but appeared to exhibit these less pronounced. Moderate increases were observed in metabolites such as p-cresol sulfate and tryptamines reflecting mild disruptions in gut microbial metabolism and inflammation. Additionally, the metabolic variations within this group indicate a potential link between energy metabolism dysfunction and chronic inflammation, factors commonly associated with ME/CFS pathophysiology.

Although overall BUM levels did not significantly differ between the groups, distinct metabolic patterns emerged due to metabolite-specific dysregulation. This discrepancy may be partly explained by heterogeneous distribution, where some metabolites are elevated in certain individuals but reduced in others, masking overall statistical significance. The heatmap clustering highlights variations in specific metabolites, suggesting selective disruptions in metabolic pathways rather than a uniform shift in BUM levels. Compensatory mechanisms may also play a role, balancing total BUM levels while still driving changes in individual metabolites. Additionally, variability in gut microbiota composition contributes to diverse metabolite levels, with some individuals exhibiting heightened microbial activity in specific pathways. Finally, BUM alterations appear to be disease-specific, as distinct clustering patterns indicate pathway-specific metabolic dysregulation in PCS and ME/CFS rather than a global shift in BUM levels.

### 3.4 Changes in metabolite composition due to symptom manifestation

As variability in metabolic profiles was observed in the heatmap analysis, we next investigated whether the presence of specific symptoms, as defined by the Canadian Consensus Criteria (CCC), was associated with changes in particular metabolites across the entire study population. Therefore, the study cohort was divided into two groups based on whether participants answered “yes” or “no” to having a certain symptom in the CCC. These two groups were then compared to determine if metabolite levels differed between participants with the symptom and those without it.

Indole-3-acetic acid and indoxyl sulfate were associated with fatigue and neurological symptoms, suggesting microbial contributions to neuroinflammation and cognitive dysfunction. Trimethylamine-N-oxide and hippuric acid are linked to neuroendocrine abnormalities, including temperature dysregulation and altered circadian rhythms, which could reflect disturbances in autonomic function and metabolic regulation. P-cresol sulfate was associated with intestinal issues and dyspnea, pointing to possible gut-lung interactions and systemic inflammatory effects. Additionally, phenylacetylglutamine correlated with vertigo, suggesting a role in autonomic dysfunction. However, after adjusting for multiple comparisons, these associations were no longer statistically significant (**Table 6**).

**Table 6:** Metabolites associated with symptoms in CCC cohorts (unadjusted and adjusted).

Category CCC	Symptom	Metabolite	p-value^1^	p-value adjusted^2^
Fatigue	Overall	Indole-3-acetic acid	**0.036**	0.325
Neuroendocrine abnormalities	Temperature dysregulation	Trimethylamine-N-Oxide	**0.014**	0.125
Neuroendocrine abnormalities	Temperature dysregulation	Hippuric acid	**0.038**	0.172
Neuroendocrine abnormalities	Heat/cold intolerance	Trimethylamine-N-Oxide	**0.011**	0.101
Autonomic symptoms	Vertigo	Phenylacetylglutamine	**0.028**	0.255
Autonomic symptoms	Intestinal Issues	P-cresol sulfate	**0.024**	0.212
Autonomic symptoms	Dyspnea	P-cresol sulfate	**0.032**	0.292
Neurological and cognitive symptoms	Overall	Indoxyl sulfate	**0.047**	0.419
Neurological and cognitive symptoms	Coordination issues	Indole-3-acetic acid	**0.007**	0.066
Sleep disturbances	Altered day-night rhythm	Trimethylamine-N-Oxide	**0.026**	0.117
Sleep disturbances	Altered day-night rhythm	Hippuric acid	**0.020**	0.117

^1^Mann-Whitney U-Test.

^2^Benjamini & Hochberg correction for multiple testing.

Significant P-values are bold.

Thus, our results in a rather small population of patients with post-infectious syndromes have to be interpreted with caution. Further research in bigger cohorts of patients is necessary to investigate the relevance of the data in more detail to better understand the impact of gut-derived metabolites on symptom severity and disease mechanisms in PCS and ME/CFS.

## 4 Discussion

This pilot study investigated concentrations of different bacterial uremic metabolites (indoxyl sulfate, indole-3-acetic acid, p-cresol sulfate, trimethylamine, trimethylamine-N-oxide, phenylacetylglutamine, tryptamine, HPHPA, and hippuric acid) in patients with post-COVID-19 syndrome (PCS) and Myalgic Encephalomyelitis/Chronic Fatigue Syndrome (ME/CFS) in comparison to a healthy control group (Ctrl). The study used metabolomic analysis, a non-invasive method for detecting diverse metabolites from a single urine sample, and examined whether physical, cognitive, psychological, somatic symptoms and trauma experience were related to concentrations of bacterial uremic metabolites (BUM).

Overall, symptom load was very high in patients with post-infectious syndromes (i.e., PCS and ME/CFS). Especially fatigue and neurological symptoms were highly expressed in many patients with PCS and ME/CFS, who reported notably higher symptom scores compared to the Ctrl group. Our data thus confirm a substantial burden of physical symptoms, which can severely impair the quality of life of patients. Patients with PCS and ME/CFS very often presented with a lot of symptoms in common, in fact, about half the PCS patients fulfilled the CCC for ME/CFS, suggesting similar underlying pathomechanisms.

Associations between symptom severity and concentrations of BUM suggest that gut dysbiosis might play a role in the development of these conditions, warranting further investigation of the role of gut microbiota in post-infectious syndromes. Gut dysbiosis has been proposed earlier to play an important role in the pathogenesis of PCS (Zhang F. et al., 2023), however, to our knowledge no associations between symptoms of patients and BUM have been described in patients with PCS previously. We found elevated concentrations of BUM in a high percentage of patients with PCS, and gut metabolite profiles tended to differ between individuals with post-infectious fatigue (i.e., PCS and ME/CFS) and healthy Ctrls. Certain metabolites like HPHPA, trimethylamine and hippuric acid were higher than reference ranges only in patients with PCS, indicating that they might be potential biomarkers for PCS. Tryptamine was elevated only in patients with both PCS and ME/CFS, but not in Ctrls, and might thus also be suited as a marker for post-infectious syndromes. On the other hand, it is important to interpret the findings of our pilot study with caution: the fact that the concentrations of all these BUM did not differ significantly between patients with PCS, ME/CFS, and Ctrl, and the fact that associations between symptoms and metabolites were no longer significant after adjustment for multiple testing, clearly indicate that larger studies involving more patients are necessary to better characterize metabolite profiles and investigate the role of gut dysbiosis.

Still, our pilot study indicates, that the determination of bacterial metabolites in the urine can be useful to screen for gut dysbiosis and that certain BUM could also be potential biomarkers for post-infectious syndromes or for certain symptoms. As determination of BUM in urine is non-invasive and easy to perform, it could be used for analyses with more patients without any problem. In fact, biomarkers for post-infectious syndromes are urgently needed and BUM have the potential to be such biomarkers. Urinary metabolomics analysis might additionally also be useful to monitor the effects of interventions with prebiotics, probiotics, and dietary changes in patients with post-infectious syndromes, as urine samples are easier to acquire as blood samples, and urine stabilized with acid is more stable than stool samples. Unfortunately, stool microbiota analyses were not available in this pilot study, which could have provided a better understanding of how concentrations of BUM are related to the abundance of certain pathogens or commensals, and also to symptoms experienced by PCS and ME/CFS patients. Depending on the extent of dysbiosis, the specific bacterial subspecies involved, environmental factors, or variations in organ function, patients may have different symptoms with varying degrees of severity—during acute COVID-19 infection, but also thereafter.

Interestingly, an altered composition of the gut microbiota prior to COVID-19 infection has been linked to various COVID-19 symptoms, particularly those involving the gastrointestinal system, as well as lingering post-COVID-19 symptoms. Studies have shown that the initial diversity and composition of the human gut microbiome is associated with distinct clinical outcomes during and after COVID-19, varying across populations (Zhang D. et al., 2023; Zuo et al., 2023). Furthermore, research has demonstrated that transplanting gut microbiota from individuals with PCS into germ-free healthy mice induces lung inflammation in the absence of SARS-CoV-2 infection, suggesting that the intestinal microbiota of PCS patients may contribute to this condition. As these mice also presented with cognitive impairment, gut microbiota dysbiosis appears to induce both lung inflammation and neurocognitive symptoms (de Almeida et al., 2023), which would help explain why patients might have persistent symptoms.

In our pilot study, PCS went along with rather low concentrations of indoxyl sulfate and indole-3-acetic acid, while HPHPA, p-cresol sulfate and tryptamine levels were elevated more often compared to Ctrls. ME/CFS metabolic profiles showed greater heterogeneity, such as HPHPA being elevated. The metabolite patterns in PCS and ME/CFS indicate metabolome and microbiome dysregulation, possibly contributing to disease pathology. The healthy Ctrl group shows generally neutral to slightly increased levels of metabolites such as hippuric acid, indoxyl sulfate, and indole-3-acetic acid, suggesting that metabolic and microbiome functions are well-maintained.

Interestingly, increased levels of tryptamine were only found in patients with post-infectious syndromes: Tryptamine is a monoamine compound that serves as a precursor for several important neurotransmitters, including serotonin (Lanser et al., 2020). A recent publication showed various molecules that are depleted in both acute and post-acute COVID-19 conditions. Among these, serotonin stood out as the most prominent. Notably, in the post-acute phase of infection, serotonin levels proved to be indicative of whether a patient would fully recover or experience long-term sequelae. Moreover, they discovered that serotonin levels decreased in other viral infections indicating that this could be a broader trait associated with systemic viral infections in general (Wong et al., 2023). As serotonin is also called “the joy hormone” deficiencies of serotonin would also explain, why many PCS patients have depression.

Moreover, tryptamine and indole-3-acetic acid are downstream metabolites of the essential amino acid tryptophan, the availability of which in fact is closely connected to neurotransmitter synthesis of serotonin and energy production (NAD) (Gao et al., 2020). Enhanced tryptophan catabolism by gut pathogens and proteobacteria respectively, might in fact explain symptoms like fatigue, depression or sleep disturbance in patients with PCS (Eroglu et al., 2021). Interestingly, in patients with chronic kidney disease higher levels of indole-3-acetic acid led to a higher mortality rate and cardiovascular events in these patients (Dou et al., 2015). On the other hand, treatment with indole-3-acetic acid in a chronic mild stress mouse model led to a noteworthy decrease in anxiety and depression-like behaviors (Chen et al., 2022). These results would fit with the rather low indole-3-acetic acid levels in the PCS and ME/CFS study cohort. Additionally, indole-3-acetic acid has the capacity to regulate intestinal balance and inhibit inflammatory reactions. Treatment of mice with ankylosing spondylitis with indole-3-acetic acid reduced the severity of ankylosing spondylitis in mice and reduced the amount of pro-inflammatory cytokines. Most importantly, treatment with indole-3-acetic acid improved intestinal mucosal barrier function and restored balance of the intestinal microbiome (Shen J. et al., 2022).

Interestingly, indoxyl sulfate was rather low in most PCS and ME/CFS patients. Elevated levels of this uremic toxin, which is typically cleared by the kidneys and excreted in urine, has been linked to impaired cognitive function in renal dialysis patients (Lin et al., 2019). Indoxyl sulfate leads to increased oxidative stress and pro-inflammatory cytokine signaling in glial cells, suggesting a potential role for inflammation and reactive oxygen species (Adesso et al., 2017). In addition, higher levels of indoxyl sulfate correlated with increased anxiety (Brydges et al., 2021).

There is limited research specifically linking HPHPA to ME/CFS and PCS. Nevertheless, there has been research conducted on HPHPA in relation to other conditions, which present with similar symptoms. Increased levels of HPHPA have been detected in urine samples from subjects diagnosed with autism spectrum disorders and schizophrenia, suggesting a potential association with imbalances in the composition of gut microbiota. Given the established link between gut dysbiosis, HPHPA may also be relevant in this context (Shaw, 2010; Bolton and Robertson, 2016).

P-cresol sulfate levels were also elevated more often in PCS patients. Interestingly, in the context of neurological disorders, an increased urinary p-cresol sulfate has been observed in children diagnosed with autism. Furthermore, p-cresol has been demonstrated to exert an influence on brain dopamine metabolism and to promote autistic behaviors in murine models (Persico and Napolioni, 2013; Pascucci et al., 2020).

In fact, treatment of gut dysbiosis might be an interesting therapeutic option, which is already available and has been shown to be effective. A trial in Hong Kong tested the synbiotic SIM01 for treating post-acute COVID-19 syndrome in 463 patients. After six months, SIM01 significantly improved symptoms like fatigue, memory loss, and difficulty concentrating compared to placebo. These findings suggest gut microbiome modulation as a potential PCS treatment (Lau et al., 2024). However, individual differences in microbiome composition and metabolite clearance- as shown by the heatmap analysis- should probably be taken into consideration in patients with very severe and maybe also therapy-resistant symptoms. In such patients precision medicine approaches targeting gut health might be especially useful. Targeted symbiotic therapies treating underlying individual microbiome dysbalances could be more effective in alleviating symptoms and improving patient outcomes than a single probiotic treatment.

However, more research has to be performed to really assess the effects of such probiotic or symbiotic interventions longitudinally. Moreover, not only the composition of gut microbiota or probiotic treatments (which a few patients took) may have an influence on results of urine metabolomics, but also other potential confounding factors such as diet, medication use and lifestyle differences- which were not accounted for in our pilot study.

When interpreting our results also other limitations of our study need to be considered: The small sample size, particularly in the ME/CFS group, reduces statistical power and limits the specificity of our findings. In addition, the cross-sectional design does not allow us to assess, whether the observed metabolic changes are involved in the development of PCS and ME/CFS symptoms or not.

Although we implemented dietary restrictions prior to urine collection and all patients were instructed earlier to eat an “anti-inflammatory” diet (mediterranean diet, poor in refined sugar and histamine containing foods) we do not know, whether all patients followed that recommendation. The assessment of long-term dietary habits and supplement use could provide greater insight into their impact on metabolite levels. As also prior medications such as antibiotics or current medication like proton pump inhibitors (PPIs), and non-steroidal anti-inflammatory drugs (NSAIDs) can significantly alter gut microbiota composition and metabolite production (Jackson et al., 2016; Rogers and Aronoff, 2016). The influence of commonly used drugs should be carefully considered in future research to distinguish disease-related metabolic changes from medication-induced effects. Another important factor that requires further investigation is the role of hepatic metabolism in BUM clearance. The liver is responsible for metabolism and detoxification of medication, but also many bacterially derived metabolites. Liver dysfunction, whether due to systemic inflammation or direct viral effects, could affect BUM levels (Vanholder et al., 2003; Snauwaert et al., 2024).

Also kidney function might affect BUM concentrations. Although none of the participants in our study had a diagnosed kidney disease, ultimately only results of 41 out of 56 patients (73%) could be included, as the creatinine levels of the other participants fell outside the specified reference ranges. Only individuals with urine creatinine levels within the reference range were included to prevent potential dilution effects or falsely elevated concentrations from impacting the results. The exclusion of such a significant proportion of patients was unexpected. However, given the established correlation between metabolite concentrations and creatinine levels (Biovis Lab), we included only patients with levels within the reference range to avoid bias, as impaired renal clearance can influence BUM concentrations (Evenepoel et al., 2009).

Furthermore, a potential selection bias should be considered, as participants were recruited from a single clinical site, potentially limiting the generalizability of findings to broader PCS and ME/CFS populations. Additionally, the inclusion criterion of a PCFS score ≥2 may have excluded milder PCS cases, thereby focusing primarily on individuals with moderate to severe functional impairment. Given the known sex differences in microbial metabolism and immune responses, the higher proportion of female participants in the study should also be considered when interpreting findings. In general, women are more often affected by PCS, which is clearly also shown in this study cohort (Bai et al., 2022). Future research should investigate potential sex-based differences in BUM metabolism and explore whether findings can be generalized to a more diverse cohort. Moreover, potential detection bias may arise from the reliance on self-reported symptom severity measures, though validated clinical criteria were used to minimize subjectivity. Additionally, urine sample processing was standardized to reduce variability in metabolite stability.

To conclude, our pilot study indicates that gut dysbiosis, reflected by altered concentrations of certain bacterial uremic metabolites, may contribute to the pathophysiology of PCS and ME/CFS. Distinct metabolic patterns between PCS and ME/CFS patients compared to healthy Ctrls suggest potential links between microbiome dysregulation and symptom severity. Despite the study’s limitations the results of our pilot study are promising and might pave the way for bigger studies with more PCS and ME/CFS patients. Larger, longitudinal studies that incorporate microbiota analysis, dietary factors, and a broader participant demographic will hopefully lead to a better understanding of the role of BUM and gut dysbiosis in patients with post-infectious syndromes.

## Data Availability

The original contributions presented in the study are included in the article/supplementary material. Further inquiries can be directed to the corresponding authors.
